# Supplementary material for: Automated evaluation can distinguish the good and bad AI responses to patient questions about hospitalization
Source: NPJ Digit Med. 2026 May 30;9:656. doi: 10.1038/s41746-026-02727-x (PMC13518972; doi:10.1038/s41746-026-02727-x)
Supplement: Supplementary file 1 [file 41746_2026_2727_MOESM1_ESM.pdf]

Supplementary Materials for “Automated Evaluation can Distinguish the Good and Bad AI Responses to Patient Questions about Hospitalization”

1. Annotation Guidelines

1.1 Evaluation Criteria Descriptions

Criterion 1: Does the output answer the question?

Rating	Description
Yes	Output includes a full, direct, and explicit answer to the provided question
Partially	Output includes only a partial answer or implies but does not directly state the answer
No	Output does not answer question directly or indirectly

Criterion 2: Does the output use the EHR evidence? And how?

Rating	Description
Yes	At least one assertion is supported by or inferred from the evidence
No	No assertions in the output use the evidence
Refutes	At least one assertion is refuted by the evidence

Criterion 3: Does the output use any general knowledge?

Rating	Description
Yes	Uses general knowledge that is in line with or neutral to the question
No	Does not include any general knowledge
Conflicting	Provides contradictory or incorrect knowledge (including hallucinations)

1.2 Evaluation Criteria Examples

Criterion 1: Does the output answer the question?

Rating: Yes

Question: Could this be a side effect of the medication?

Answer: 'Yes, fatigue is a known side effect of this medication class, particularly during the first few weeks of use.'

Reason: Directly answers the user's concern with specificity.

Rating: Partially

Question: Should I be worried about these symptoms?

Answer: 'Sometimes these symptoms occur with mild infections.'

Reason: Vague response, doesn't directly address the user's scenario.

Rating: No

Question: Is this a normal recovery timeline?

Answer: 'Staying hydrated is important after any illness.'

Reason: Irrelevant and generic advice.

Criterion 2: Does the output use the EHR evidence? And how?

Rating: Yes

Note: 'Patient had low-grade fever and stiff neck.'

Answer: 'Given the low-grade fever and stiff neck noted at admission, this could be consistent with viral meningitis.'

Reason: Uses note directly to justify reasoning.

Rating: No

Note: 'Mild dehydration, patient discharged.'

Answer: 'Consult your doctor if you have any symptoms.'

Reason: Does not use any information from the note.

Rating: Refutes

Note: 'No signs of cardiac involvement noted.'

Answer: 'This might be heart-related and should be urgently checked.'

Reason: Contradicts provided evidence.

Criterion 3: Does the output use any general knowledge?

Rating: Yes

Answer: 'Children often recover from viral illnesses within 7-10 days, especially with supportive care like rest and hydration.'

Reason: Useful, correct general knowledge.

Rating: No

Answer: 'Patient had fever and sore throat. These are the main symptoms noted.'

Reason: Only restates evidence with no external knowledge.

Rating: Refutes

Answer: ‘It's not uncommon for someone with viral meningitis to become infectious again several weeks after initial recovery.'

Reason: Incorrect general knowledge contradicting standard facts and EHR information.

1.3 Gold Standard Example

Question

A friend went to the emergency room this past Monday morning with severe flu-like symptoms. On Thursday, she was diagnosed with meningitis. On Friday afternoon, it was determined to be viral, and she was rereleased with instructions to stay home and rest for 1 week. Yesterday she was beginning to feel better, but is not feeling as well today as yesterday. Is this fall back today a normal part of the recovery process, or is it a sign of a possible relapse? Should she perhaps be gotten back to the hospitals? Thanks for your time.

Note Excerpt

___ with h/o hypothyroidism secondary to Hashimoto's, Chiari malformation s/p posterior fossa decompression surgery ___, h/o aseptic meningitis ___ p/w meningitis

Meningitis:

Pt. presented with headaches, neck stiffness, and fevers.

Given concern for meningitis, she was initially started on vanc/ctx/acyclovir.

Encephalitis was unlikely as patient had normal mental status.

___ LP on ___ showed WBCs 130s (70% lymphocytes).

CSF HSV PCR was negative, and her blood Lyme serologies were negative.

It was concluded that she had aseptic meninigitis, and she was taken off all antibiotics and antivirals by discharge.

She received IV dilaudid 0.5mg, ibuprofen, and tylenol for pain control in the hospital.

She was discharge home on PO dilaudid ___ Q4H as needed.

3. HYDROmorphone (Dilaudid) ___ mg PO Q4H:PRN head pain

Please take ___ mg as needed. Do not take more than 24mg each day.

RX *hydromorphone 2 mg ___ tablet(s) by mouth every 4 hours Disp #*24 Tablet Refills:*0

4. Acetaminophen 650 mg PO Q6H:PRN pain
Do not exceed 3gm/day.

5. Ibuprofen 400 mg PO Q6H:PRN pain

6. Ondansetron 4 mg PO Q8H:PRN nausea
RX *ondansetron 4 mg 1 tablet(s) by mouth every 8 hours Disp #*12 Tablet Refills:*0

___ were admitted to the hospital for fevers and neck stiffness.
___ were found to have meningitis, likely caused by a virus.
We gave ___ acyclovir, vancomycin, and ceftriaxone initially.
Your lab tests and lumbar puncture results ruled out bacterial, HSV, and Lyme meningitis.

___ were started on a new medication, dilaudid, for pain control.
___ were also on zofran for nausea.
___ can use these medications as needed.

Answer

Based on the discharge summary excerpt, it is expected that you may experience a worsening of symptoms due to viral meningitis, which can have a fluctuating course. However, if you are experiencing severe symptoms or concerns, it's essential to contact your healthcare provider for further evaluation and guidance.

Evaluation Criteria

Criterion 1: Does the output answer the question?

Rating: Yes

Explanation:

The answer directly and fully addresses the user's concern. It explains that fluctuating symptoms can be typical during viral meningitis recovery, while also advising the person to seek medical attention if severe symptoms occur. This makes the answer complete, relevant, and clear.

Criterion 2: Does the output use the EHR evidence? And how?

Rating: Refutes

Explanation:

The answer says patient has viral meningitis, whereas the note clearly states it was ruled out and the final diagnosis is aseptic meningitis.

Criterion 3: Does the output use any general knowledge?

Rating: No

Explanation:

The answer doesn't add any outside medical information. It doesn't reference clinical guidelines, recovery timelines, or data from medical studies. Instead, it only makes general comments based on the note, like saying symptoms might change and to talk to a doctor if needed. So, the answer sticks to what's in the note and doesn't bring in extra medical knowledge.

2. System-by-system results using automated metrics

Table S1. Automated evaluation scores for the 28 answer-generation systems evaluated in this study. Systems are ranked by **Overall score** (**O**), **Factuality** (**F**; strict macro citation F1), and **Relevance** (**R**; average relevance computed with clinician authored reference answers). **O** (and **R**) use the clinician authored-reference scores; note-reference relevance scores are reported for comparison and yield a separate overall score column. Factuality scores report macro-averaged **Precision (P)**, **Recall (R)**, and **F1** under **strict** (only essential sentences treated as relevant) and **lenient** (essential+supplementary) sentence relevance definitions. Relevance is computed against two reference types: **Relevance (human)** uses clinician authored reference answers, while **Relevance (note)** uses a proxy reference formed by concatenating note sentences labeled **essential** with the input question. Relevance metrics include text-overlap scores (**BL**=BLEU, **RG**=ROUGE, **SA**=SARI) and semantic scores (**BS**=BERTScore, **AS**=AlignScore, **MD**=MEDCON); **Avg** is the mean relevance across the listed relevance metrics for the given reference type. **OS** denotes the overall score, defined as the mean of **strict** factuality F1 and the corresponding **Avg** relevance score (reported separately for human vs. note references). All scores are percentages.

				Factuality						Relevance (human)								Relevance (note)							
Rank			System	Lenient			Strict			Text			Semantics					Text			Semantics				
O	*F*	*R*	ID	P	R	F1	P	R	F1	BL	RG	SA	BS	AS	MD	Avg	OS	BL	RG	SA	BS	AS	MD	Avg	OS
1	3	2	M1	78.3	67.2	68.4	56.7	75.5	61.9	**8.4**	**24.8**	**55.9**	40.8	36.9	**43.2**	<u>35.0</u>	**48.5**	7.5	34.6	67.1	38.4	58.7	<u>45.9</u>	42.0	52.0
2	4	1	M2	81.8	52.5	58.7	64.3	60.8	59.8	7.4	<u>24.6</u>	53.9	40.2	**47.9**	41.6	**35.9**	<u>47.9</u>	2.7	28.0	56.0	30.8	53.9	39.8	35.2	47.5
3	1	7	M3	80.2	59.8	65.2	60.7	69.1	**62.3**	4.2	21.3	53.0	41.0	34.6	41.9	32.7	47.5	1.7	24.8	54.7	30.6	53.0	41.7	34.4	48.3
4	2	22	M4	76.5	<u>75.6</u>	**72.3**	54.6	**82.8**	<u>62.1</u>	6.3	20.7	53.1	30.6	25.9	33.3	28.3	45.2	<u>11.5</u>	<u>36.0</u>	**73.9**	40.0	61.1	40.4	43.8	<u>52.9</u>
5	8	7	M5	84.6	47.5	55.9	<u>67.0</u>	55.2	56.8	<u>8.1</u>	24.1	54.3	36.8	33.7	39.3	32.7	44.8	7.5	35.5	**73.9**	<u>41.0</u>	<u>71.2</u>	42.0	<u>45.2</u>	51.0
6	6	14	M6	78.6	61.5	64.4	55.5	67.2	57.5	5.5	22.2	54.3	38.6	25.7	39.3	30.9	44.2	3.7	27.8	63.8	32.5	48.8	40.0	36.1	46.8
6	11	3	M7	**86.8**	43.7	54.1	**68.7**	51.1	54.5	4.5	22.8	<u>54.5</u>	<u>41.7</u>	<u>40.5</u>	38.9	33.8	44.2	1.0	21.7	57.6	27.9	56.9	34.5	33.3	43.9
8	9	7	M8	<u>84.7</u>	43.7	53.6	66.8	51.3	55.0	6.7	23.6	51.7	40.3	36.0	38.1	32.7	43.9	1.9	25.3	51.4	28.3	53.5	34.9	32.6	43.8
9	5	17	M9	73.3	74.3	<u>68.9</u>	51.9	<u>79.9</u>	57.8	7.4	22.0	53.3	33.9	26.8	34.4	29.6	43.7	8.6	35.0	<u>71.2</u>	38.0	59.1	39.0	41.8	49.8
10	12	6	M10	80.7	47.9	56.2	58.5	53.8	53.4	7.1	23.4	54.1	39.3	35.0	39.1	33.0	43.2	4.0	29.8	65.4	34.5	59.3	40.8	39.0	46.2
10	15	3	M11	77.9	45.6	53.4	60.3	51.6	52.6	6.8	24.2	53.1	**42.5**	35.8	40.2	33.8	43.2	1.6	24.9	54.1	29.0	52.2	37.9	33.3	42.9
12	13	12	M12	81.1	39.0	48.8	<u>67.0</u>	47.5	53.1	5.5	22.7	49.2	40.2	35.6	38.3	31.9	42.5	1.8	25.1	48.8	28.6	55.0	36.9	32.7	42.9
13	16	10	M13	68.9	46.5	51.0	53.6	54.4	52.0	5.2	21.7	51.6	38.7	39.9	37.8	32.5	42.3	2.3	25.5	49.8	27.1	49.0	33.8	31.3	41.6
14	17	12	M14	67.4	64.9	62.0	48.0	69.0	51.9	5.5	23.4	53.8	38.2	27.8	<u>42.8</u>	31.9	41.9	2.4	24.3	59.8	29.3	50.1	38.3	34.0	43.0
15	10	20	M15	72.5	56.6	59.3	54.5	64.3	54.8	4.4	19.1	52.3	31.6	30.7	34.8	28.8	41.8	5.7	27.7	64.6	33.1	56.2	39.0	37.7	46.2
16	20	11	M16	72.5	43.2	49.5	54.1	48.9	48.4	5.1	22.9	53.5	40.8	31.2	39.8	32.2	40.3	0.9	21.7	51.0	25.5	41.1	34.3	29.1	38.7
17	18	16	M17	63.8	58.0	56.6	45.1	63.8	50.6	4.3	23.2	53.4	39.5	23.3	34.5	29.7	40.2	0.9	21.6	56.9	25.8	34.7	30.0	28.3	39.5
18	14	25	M18	68.4	71.1	65.6	45.8	74.4	52.8	2.9	18.4	48.5	34.9	25.8	32.8	27.2	40.0	1.1	23.1	49.1	24.3	41.8	33.1	28.8	40.8
19	19	23	M19	59.0	73.9	62.4	40.1	78.4	49.9	6.2	21.8	50.6	31.5	24.3	34.3	28.1	39.0	4.7	28.1	55.6	28.0	40.8	33.6	31.8	40.9
20	23	5	M20	77.4	34.4	43.2	61.2	39.0	44.2	5.7	23.1	51.8	40.2	38.3	39.9	33.2	38.7	1.5	22.9	53.1	27.2	52.7	35.2	32.1	38.1
21	21	19	M21	68.7	47.6	51.4	51.5	52.7	47.8	2.8	20.6	51.3	38.5	28.5	33.4	29.2	38.5	0.8	21.3	48.9	25.1	41.2	32.5	28.3	38.1
22	22	26	M22	56.4	**80.3**	61.7	35.5	79.8	46.2	2.5	17.5	51.5	33.9	22.7	30.9	26.5	36.4	1.1	19.4	57.9	24.4	36.8	31.1	28.5	37.4
23	7	28	M23	73.9	70.3	68.6	50.5	74.7	57.2	0.6	12.7	34.8	19.1	9.2	16.2	15.4	36.3	**18.6**	**52.4**	36.2	**57.8**	**91.2**	**54.0**	**51.7**	**54.5**
24	24	21	M24	63.9	41.3	45.4	45.6	44.5	41.1	4.6	21.0	48.6	37.3	26.5	33.2	28.5	34.8	2.4	25.7	47.4	26.1	43.6	36.4	30.3	35.7
25	25	18	M25	68.1	32.3	38.3	51.1	34.6	37.6	5.1	21.3	49.8	37.2	27.9	35.1	29.4	33.5	2.1	25.3	50.7	28.1	45.7	36.5	31.4	34.5
25	26	15	M26	82.4	24.5	32.9	66.3	28.5	36.7	4.4	18.7	51.5	36.1	37.4	33.6	30.3	33.5	1.0	20.0	54.3	24.7	59.1	31.6	31.8	34.2
27	27	27	M27	62.9	34.8	40.8	39.7	35.1	35.3	4.2	18.8	51.7	29.3	24.7	26.0	25.8	30.6	2.1	23.4	61.5	28.8	52.0	26.3	32.4	33.8
28	28	24	M28	57.8	13.4	18.3	47.4	14.6	20.2	2.9	19.4	48.7	32.7	29.4	31.9	27.5	23.8	0.9	18.6	47.2	21.8	44.1	27.3	26.6	23.4
